# Appraisal of Clinical Explanatory Variables in Subtyping of Type 2 Diabetes Using Machine Learning Models

**DOI:** 10.3390/jcm14186548

**Published:** 2025-09-17

**Authors:** Amar H. Khamis, Fatima Abdul, Stafny Dsouza, Fatima Sulaiman, Costerwell Khyreim, Mohammed E. Siddig, Riad Bayoumi

**Affiliations:** 1 Hamdan Bin Mohammed College of Dental Medicine, Mohammed Bin Rashid University of Medicine and Health Sciences, Dubai P.O. Box 505055, United Arab Emirates; 2 College of Medicine, Mohammed Bin Rashid University of Medicine and Health Sciences, Dubai P.O. Box 505055, United Arab Emirates; fatima.abdul@dubaihealth.ae (F.A.); stafny.dsouza@dubaihealth.ae (S.D.); fatima.sulaiman@students.mbru.ac.ae (F.S.); costerwell.khyriem@dubaihealth.ae (C.K.); 3 College of Science, University of Gezira, Wad Madani P.O. Box 20, Gezira, Sudan; mohdnoor@softcareez.com

**Keywords:** artificial intelligence, type 2 diabetes, subtyping, aetiology, logistic regression

## Abstract

**Background**: Clustering type 2 diabetes (T2D) remains a challenge due to its clinical heterogeneity and multifactorial nature. We aimed to evaluate the validity and robustness of the clinical variables in defining T2D subtypes using a discovery-to-prediction design. **Methods**: Five explanatory clinical aetiology variables (fasting serum insulin, fasting blood glucose, body mass index, age at diagnosis and HbA1c) were assessed for clustering T2D subtypes using two independent patient datasets. Clustering was performed using the IBM-Modeler Auto-Cluster. The resulting cluster validity was tested by multinomial logistic regression. The variables’ validity for direct unsupervised clustering was compared with machine learning (ML) predictive models. **Results**: Five distinct subtypes were consistently identified: severe insulin-resistant diabetes (SIRD), severe insulin-deficient diabetes (SIDD), mild obesity-related diabetes (MOD), mild age-related diabetes (MARD), and mild early-onset diabetes (MEOD). Using all five variables yielded the highest concordance between clustering methods. Concordance was strongest for SIRD and SIDD, reflecting their distinct clinical signatures in contrast to that in MARD, MOD and MEOD. **Conclusions**: These findings support the robustness of clinically defined T2D subtypes and demonstrate the value of probabilistic clustering combined with ML for advancing precision diabetes care.

## 1. Introduction

Type 2 diabetes (T2D), the 8th leading cause of global disease burden affecting more than 500 million individuals, is estimated to become the second leading cause by 2050 [1]. However, it remains difficult to characterize due to its heterogeneity, arising from diverse pathophysiological mechanisms—including varying degrees of insulin resistance, beta-cell dysfunction, and metabolic disturbances. This variability is further complicated by overlapping comorbidities such as obesity, hypertension, and cardiovascular disease. Although these factors make it difficult to clearly distinguish subtypes, precise classification remains essential for enabling personalized treatment strategies and improving the prediction of long-term complications.

Clustering approaches have been central to efforts to dissect this heterogeneity utilizing clinical and genetic variables. Ahlqvist et al. [2] identified five reproducible clusters of diabetes by K-means based on six clinical variables: age at diagnosis, body mass index (BMI), glycated haemoglobin (HbA1c), homeostatic model assessment for beta cell dysfunction (HOMA2-B), homeostatic model assessment for insulin resistance (HOMA2-IR), and glutamic acid decarboxylase antibody (GADA). These clusters demonstrated differing risks for complications and clinical outcomes and have since been replicated wholly or partially across multiple populations, including cohorts in Europe [2,3,4,5,6,7,8], Asia [9,10,11,12,13,14] and the Middle East [15,16]. We have previously replicated four clusters [17] termed severe insulin-resistant diabetes (SIRD), severe insulin-deficient diabetes (SIDD), mild obesity-related diabetes (MOD), and mild age-related diabetes (MARD), along with a novel South-Asian specific mild early-onset diabetes (MEOD). Genetic clustering [18,19] has added further granularity to T2D stratification. An extension of Udler’s soft clustering framework [19] to identify genetically informed clusters that highlight causal pathways of T2D heterogeneity, identified 12 genetic clusters in alignment with clinical stratification across diverse populations highlighting ancestry-specific differences [20]. Studies have also confirmed the reproducibility of these clusters with an emphasis on the emergence of new subgroups when incorporating additional biomarkers such as lipids or inflammatory markers [21,22].

Despite these advances, important gaps persist. Traditional clustering methods such as k-means often perform poorly in high-dimensional or noisy datasets, and hard assignment of individuals to a single cluster may not reflect the reality of overlapping or shared pathophysiological processes [17]. Only a few studies have identified the overlap between the T2D subtypes in European [19,23,24,25] and Arab [17] ancestry. A movement of patients between clusters over time has also been suggested showing varying outcomes across populations highlighting population-specific differences [6,25]. While probabilistic and soft clustering approaches have been proposed, they remain underutilized in clinical research, particularly in integrating both clinical and genetic information. Moreover, most studies to date have emphasized cluster discovery rather than validating the explanatory power of individual clinical variables or comparing the performance of unsupervised clustering with cluster-based supervised predictive models. Population-specific variability further complicates generalizability, with clusters defined in European cohorts not always transferring effectively to other ancestries. These limitations highlight the need for more sophisticated, flexible, and biologically informed models to better capture the complexity of T2D.

In this study, our aim was to evaluate the validity and robustness of five key variables (fasting serum insulin, fasting blood glucose, body mass index, age at diagnosis and HbA1c) in defining T2D subtypes while addressing key methodological gaps. We compared the explanatory power of these variables using direct supervised clustering and machine learning approaches [7,12,26,27], which, unlike traditional k-means, accommodate soft membership and capture overlapping disease processes [17,19,23,25]. Extending beyond prior within-cohort studies, we tested reproducibility across two distinct cohorts differing in ethnicity, disease duration, and clinical context. We further evaluated how different combinations of clinical variables influenced clustering stability, and quantified reproducibility using both Adjusted Rand Index and Fowlkes–Mallows Index, providing rigorous dual metrics of stability. By examining patterns of overlap and cluster membership under these conditions, our study offers new insights into the continuum of T2D phenotypes and lays the foundation for subtype-specific strategies in precision medicine.

## 2. Materials and Methods

### 2.1. Study Design

The study was carried out in two phases. The first phase focused on examining the use and impact of explanatory variables for clustering of type 2 diabetes (T2D). In the second phase, ML was used to test the difference between direct clustering versus cluster-based classification using predictive models employing the same sets of variables.

### 2.2. Patients

The study was conducted in the selected healthcare facilities of Dubai Health, an integrated academic health system recently established in Dubai, United Arab Emirates (UAE). It included two tertiary healthcare centers (THC)—Dubai Hospital and the Dubai Diabetes Centre (DDC)—26 primary healthcare centers (PHCs), and Mohammed Bin Rashid University (MBRU). The two independent datasets, stratified by disease duration, were generated. For each patient, the clinical and laboratory data were extracted from the SALAMA electronic health record system, implemented across all Dubai Health facilities. Only participants with no missing data were included.

Dataset 1 (training cohort): 348 Emirati patients with long-standing T2D (mean duration 14 years), each with ≥2 complications, 3–4 medications, recruited from tertiary centres between January 2020 and December 2022 [17].

Dataset 2 (validation cohort): 586 multi-ethnic patients with newly diagnosed T2D (mean duration 4 years), no comorbidities or complications, 0–2 medications, enrolled at PHCs between January 2022 and December 2023.

Models were developed in Dataset 1 and externally validated in Dataset 2, which differed by ethnicity, disease duration, complication burden, and care setting. This design ensured that generalizability was tested across heterogeneous patient populations and clinical environments.

### 2.3. Clustering Techniques

Clustering was performed using the Auto-Cluster procedure in IBM SPSS Modeler (version 18.0; IBM North America, New York, NY, USA), a data science and machine learning (ML) platform incorporating deep learning and artificial neural networks. The Auto-Cluster procedure integrates multiple algorithms, including two-step clustering (data condensation followed by hierarchical clustering), k-means clustering, and the Kohonen self-organizing neural network [17]. Models were generated using the target variable “cluster” and combinations of selected “explanatory variables” and were evaluated against multiple fit indices, with the Silhouette index serving as the primary criterion for cluster separation. Logistic regression was used to validate the clustering methods as well as to demonstrate the overlap between clusters. This approach yielded probabilistic (soft) assignments of each patient to clusters, avoiding forced hard classifications and better capturing the heterogeneity of T2D.

Model performance was further evaluated with multinomial logistic regression, treating cluster membership as the outcome and five predictors—age at diagnosis, body mass index (BMI), fasting blood glucose (FBG), fasting serum insulin (FSI), and glycated haemoglobin (HbA1c)—as independent variables. This quantified the relative contribution of each predictor to distinguishing clusters, providing an additional measure of construct validity for the clustering approach. The SPSS Modeler software (version 18.0; IBM North America, New York, NY, USA), widely applied in prior studies [17,27,28,29,30,31], sequentially tests up to 14 potential predictive models [C5.0 Decision Tree Algorithm (C5), Logistic Regression (LR), Bayesian Network (BN), Linear Discriminant Analysis (D), Linear Support Vector Machine (LSVM), Random Tree (RT), Extreme Gradient Boost Linear (XGBL), Extreme Gradient Boost Tree (XGBT), Chi-Square Automatic Interaction Detection (CHAID), Quick Unbiased Efficient Statistical Tree (QUEST), Classification and Regression Tree (C&R Tree), Neural Network (NN), Decision List (DL), Tree AS (Tree AS)] and automatically selects the top five to six based on area under the curve (AUC), silhouette index, and percent accuracy. Thus, offering significant advantages over manual model selection by efficiently handling large-scale datasets ensuring standardization, reduces subjectivity, and enhances reproducibility across analyses.

The resulting clusters were defined based on their clinical characteristics as described in Bayoumi et al. [17]. Briefly, the cluster with highest HOMA-IR, obesity and no β-cell deficiency was labelled as severe insulin-resistant diabetes (SIRD); the cluster with lowest HOMA-B and highest HbA1c as severe insulin-deficient diabetes (SIDD); the cluster with highest BMI as mild obesity-related diabetes (MOD); the cluster with highest age at diagnosis, moderate insulin resistance and no beta-cell deficiency as mild age-related diabetes (MARD); and the cluster with lower age of diagnosis, normal/overweight BMI, moderate insulin resistance and beta-cell deficiency as mild early-onset diabetes (MEOD).

#### Validation of Explanatory Variables

To validate the selection of explanatory variables for clustering, we examined four scenarios: *Scenario 1*: Included only the three fundamental etiological variables—FBG, FSI, and BMI. Unlike most previous studies, we excluded HOMA-IR and HOMA-B, as both are derived directly from FBG and FSI, thereby introducing redundancy and potential multicollinearity. This decision was supported by statistical evidence. Specifically, we evaluated collinearity using the Variance Inflation Factor (VIF) and examined the correlation structure. The VIF was calculated as

$$VIF=\frac{1}{1-R_{i}^{2}}$$

where $R_{i}^{2}$, is a coefficient of determination from the regression.

The VIF analysis demonstrated that HOMA-IR introduces severe multicollinearity when used alongside FBG and FSI with VIF = 14.1 (Supplementary Method S1). Since over 90% of its variance is explained by these two variables, HOMA-IR does not provide independent explanatory value. Including it in clustering models would outweigh glycaemic and insulin measures, bias cluster separation, and reduce interpretability.

*Scenario 2*: Added age at diagnosis (a proxy for disease chronicity) to the three basic variables.

*Scenario 3*: Added HbA1c (a marker of disease severity) to the three basic variables.

*Scenario 4*: Included all five variables—FSI, FBG, BMI, age at diagnosis, and HbA1c.

**Figure 1:**
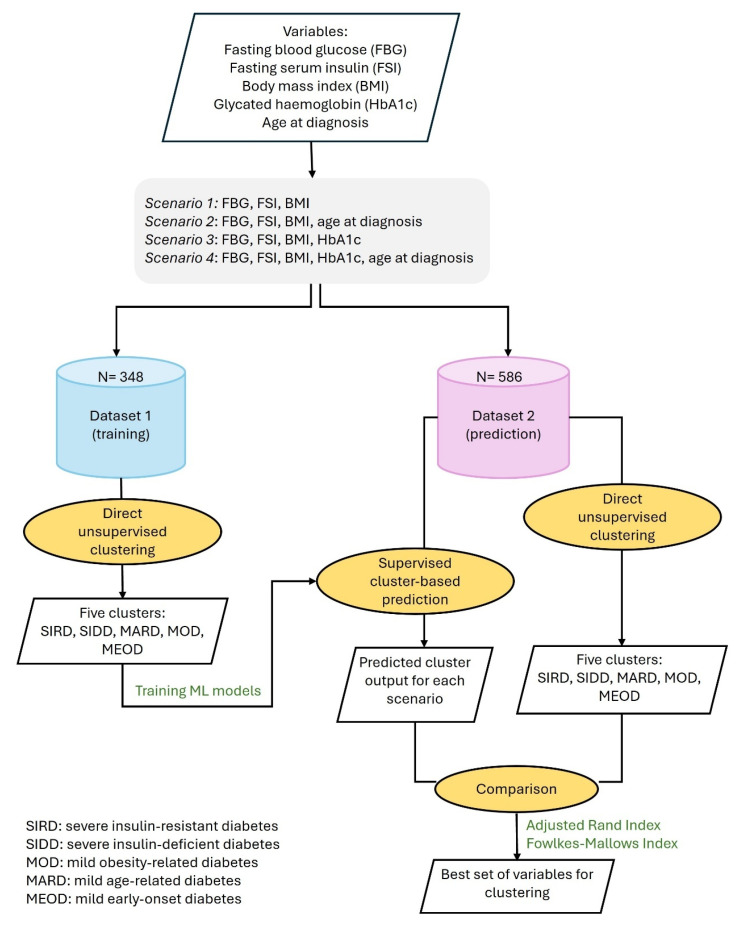
Data analysis outline.

Analysis was conducted as outlined in Figure 1. Briefly, the ML models were trained on previously reported direct clusters produced with Dataset 1 [17] and applied to Dataset 2 for supervised cluster-based prediction across all four scenarios. For comparison, Dataset 2 was also directly clustered by unsupervised methods under the same four scenarios.

### 2.4. Testing Similarity Between Clusters

Two methods were employed to assess the similarity between clusters generated by direct clustering versus cluster-based prediction using ML models. Two sets of clusters were generated for the prediction dataset: one using an unsupervised direct clustering approach and the other using a supervised cluster-based classification approach based on the previously identified unsupervised clusters. To assess the degree of similarity between the resulting cluster assignments, we calculated the Adjusted Rand Index (ARI) and the Fowlkes–Mallows Index (FMI). Both metrics quantify the agreement between two clustering results, with ARI adjusting for chance agreement and FMI evaluating the balance between precision and recall of cluster pair assignments. This analysis focuses on comparing the outputs of the two clustering approaches, rather than evaluating the intrinsic performance of the methods themselves.

#### 2.4.1. The Adjusted Rand Index (ARI)

ARI, a metric used to measure the similarity between two data clustering’s. It is commonly applied in statistics to assess the agreement between predicted clustering and a ground-truth clustering within the same dataset, calculated as

$$ARI=\frac{RI-E}{MaxRI-E}$$

where RI is Rand index value, E is the expected value of the Rand index for random clusters and Max RI is the maximum achievable value of the Rand index. Stepwise calculations of ARI are shown in Supplementary Method S2.

#### 2.4.2. The Fowlkes–Mallows Index (FMI)

FMI is a statistical measure used to evaluate the similarity between two clustering’s. It is widely applied in clustering validation to compare the agreement between a predicted clustering and a ground-truth clustering within the same dataset. The FMI assesses similarity between two clusters by utilizing precision and recall.

$$FMI=\frac{TP}{\sqrt{\left(TP+FP)(TP+FN\right)}}$$

where TP is True Positive (pairs of points that are in the same cluster in both clusters), FP is False Positive (pairs of points that are in the same cluster in one clustering but in different clusters in the other) and FN is False Negative (pairs of points that are in different clusters in one clustering but in the same cluster in the other).

The 95% confidence interval (Supplementary Method S3) for both ARI and FMI were estimated using a custom R-code for transformation and calculation using the mathematical formula:

$$z_{95CI}=z\pm 1.96{SE}_{z}$$

where z is z value and SE_z_ is standard error in z-space.

A contingency table was used to compare the assignment of data points between the two sets of results of clusters obtained by the two different methods from the same database. Furthermore, purity measures were employed to assess the homogeneity of the clusters, specifically to determine whether the data points within a cluster predominantly belong to a single true class.

### 2.5. Statistics

Data was analysed using IBM-SPSS for Windows (version 29.0; SPSS Inc., Chicago, IL, USA). Continuous variables (FSI, FBG, BMI, etc.) were described using measures of central tendency and dispersion or medians and interquartile ranges, depending on the data distribution. Categorical variables, such as clusters and overlap status, are presented as frequency and proportion. The Kolmogorov–Smirnov test was used to assess the normality of continuous variables. Mann–Whitney *U* test was used to compare the means between two groups. Chi-square test was used to assess the dependency between categorical variables (cluster and overlap). R [32] was used to calculate the Adjusted Rand Index (ARI) and Fowlkes–Mallows Index (FMI). A *p*-value of less than 0.05 was considered significant in all the statistical analyses.

## 3. Results

The key pathophysiological characteristics of the training and prediction datasets are shown in Table 1. The average primary and secondary clinical variables used for clustering differed significantly between the two datasets, except for BMI.

**Figure 2:**
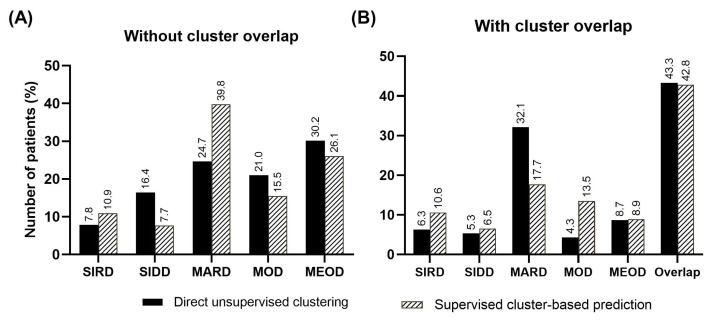
The comparison of the distribution of clusters using direct unsupervised clustering and supervised cluster-based prediction, both without cluster overlap (**A**) and with cluster overlap allowed (**B**). Clusters with overlap were identified by logistic regression. The bar plots show the percentage distribution of patient cohort (*y* axis) in five T2D subtypes (*x* axis). The clusters are labelled as severe insulin-deficient diabetes (SIDD), severe insulin-resistant diabetes (SIRD), mild age-related diabetes (MARD), mild obesity-related diabetes (MOD), mild early-onset diabetes (MEOD).

The direct clustering of the prediction dataset (*n* = 586 T2D patients) with all five exploratory variables resulted in five subtypes of T2D similar to those reported previously in the training dataset [17] in differing proportions (Figure 2). For SIRD, the proportion of patients remained low for both methods (6.3% direct clustering, 10.6% supervised prediction), consistent with the no-overlap analysis. In SIDD, 16.4% of patients were assigned to the cluster by unsupervised clustering compared to 7.7% by supervised prediction. The supervised method identified fewer patients in MARD (17.7%) when overlaps are allowed. Contrastingly, supervised prediction captured more MOD patients when overlap was permitted (13.5% vs. 4.3%). MEOD showed relatively similar proportions between methods, with more patients having overlapping features. Although a substantial proportion of patients were assigned to multiple clusters—43.3% by direct clustering and 42.8% by supervised prediction, the proportion of cluster overlap was not significant (*p* = 0.99) between the two methods (Figure 2B).

Supervised cluster-based prediction with ML generated the same five clusters in three scenarios (Table 2) except *Scenario 3* (FSI, FBG, BMI, and HbA1c), which resulted in a single mixed cluster of mild forms (Table 3). It is apparent that the more variables used in clustering, the higher the concordance.

**Figure 3:**
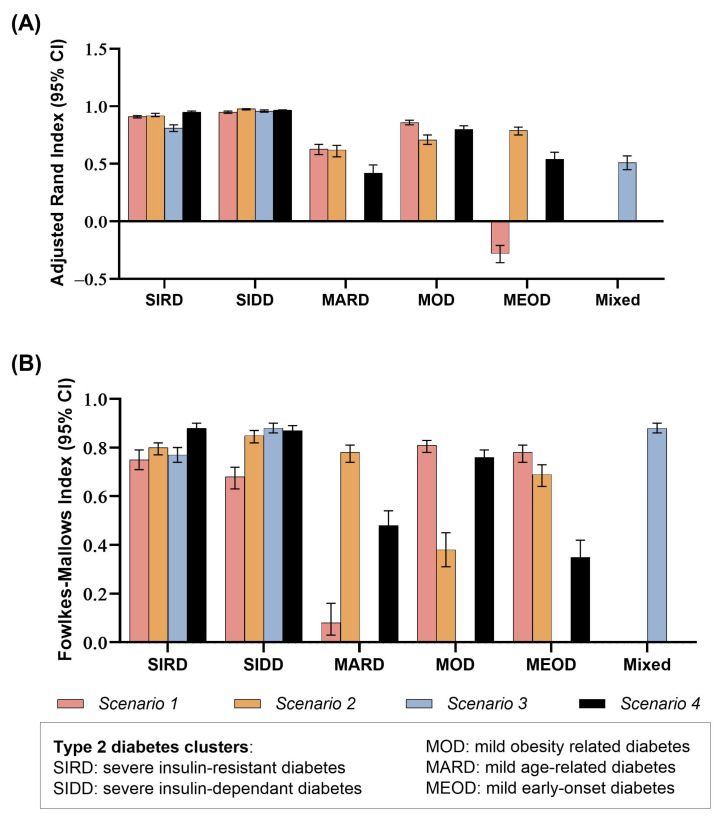
Comparison of the clustering performance by direct unsupervised clustering and supervised machine learning-based clustering with different exploratory variables: The performance was assessed for the type 2 diabetes subtypes across four scenarios of input variables (fasting blood glucose, fasting serum insulin, body mass index, glycated haemoglobin and age at diagnosis) using (**A**) Adjusted Rand Index (ARI) and (**B**) Fowlkes–Mallows Index (FMI). Bars represent mean indices, with error bars showing 95% confidence intervals. Higher values indicate stronger agreement with reference subtype assignments, reflecting greater clustering reproducibility and accuracy.

The concordance of crosstabulation of clusters was mirrored in the ARI and FMI similarity indices (Figure 3). The ARI showed high values (Supplementary Table S1) for the SIRD, SIDD clusters (>0.90) and low values for the MARD, MOD and MEOD clusters. While the MARD cluster showed weaker consistency (0.42–0.63), the MEOD cluster demonstrated unstable performance, with values ranging from poor concordance (negative ARI) to moderate (0.79). Similar results were obtained for FMI (Supplementary Table S2). Inclusion of age at diagnosis and HbA1c improved classification stability for most clusters, particularly SIRD and MARD.

## 4. Discussion

In this study, we examined the rationale for selecting clinical variables and their robustness in identifying T2D subtypes using both unsupervised and supervised machine learning (ML) clustering methods in two ethnically distinct cohorts of patients with T2D. We adopted a discovery-to-prediction design, evaluating the external validity of subtype assignment across cohorts differing in ethnicity, disease duration, and clinical context—a more stringent test than the within-cohort replications typically reported in earlier studies.

A central methodological choice in our study was the use of five key variables—fasting insulin (FSI), fasting blood glucose (FBG), body mass index (BMI), HbA1c, and age at diagnosis. Each was selected for its etiological relevance: FSI and FBG directly capture insulin resistance and β-cell dysfunction [33], BMI reflects adiposity-driven metabolic burden [34], HbA1c captures disease severity and chronicity [35], and age at diagnosis distinguishes early—from late-onset disease trajectories [36].

We deliberately excluded composite indices such as HOMA-IR and HOMA-B, despite their popularity in prior clustering studies [2,13,19]. Both indices are algebraic transforms of FBG and FSI, introducing redundancy and multicollinearity. Variance Inflation Factor analyses confirmed problematic inflation when HOMA indices were included [37,38]. By retaining the primary measures (FSI and FBG) and excluding HOMA, we improved model parsimony, stability, and biological interpretability. Importantly, once subtypes were established, HOMA indices remained useful descriptors of underlying metabolic differences, consistent with prior observations [39]. Other variables such as duration of diabetes, lipid parameters, and inflammatory markers have also been used in clustering but are either downstream consequences of disease or not directly aetiological and are therefore unsuitable for clustering inputs [13,40,41,42,43].

Using all five explanatory variables yielded the highest concordance between direct clustering and ML-based classification. We also quantified reproducibility using the Adjusted Rand Index (ARI) and the Fowlkes–Mallows Index (FMI). Strong concordance was observed for severe insulin-resistant diabetes (SIRD) and severe insulin-deficient diabetes (SIDD), whereas concordance for mild obesity-related diabetes (MOD), mild age-related diabetes (MARD) and mild early-onset diabetes (MEOD) was weaker and at times approached randomness (with near-zero or negative ARI values). Quantifying concordance is critical, as prior studies have largely inferred it qualitatively.

The severe T2D subtypes—SIDD and SIRD—emerged as the most stable clinical entities, exhibiting distinct clinical features, high concordance across methods, and minimal overlap with other subtypes, particularly in unsupervised clustering [17,25]. Their distinct metabolic signatures likely explain why these subtypes consistently replicate across populations and methods. SIDD is characterised by profound β-cell dysfunction, earlier age of onset, and poor glycaemic control [2,5,6,13,14], while SIRD is characterised by marked insulin resistance and strong associations with obesity, nephropathy, fatty liver, and cardiovascular disease [2,5,6,13,14,44,45].

By contrast, the mild forms of T2D—MARD, MOD, and MEOD—displayed weaker reproducibility, with lower concordance across methods and greater overlap with other subtypes, particularly in unsupervised clustering [17,25]. MARD, defined primarily by older age and modest dysglycaemia, was particularly unstable, with considerable overlap with MOD and MEOD. This instability suggests that MARD may encompass multiple overlapping phenotypes, consistent with evidence that older adults with T2D often exhibit blended features of insulin resistance and age-related β-cell decline [36]. MOD and MEOD also showed low concordance between methods, reflecting heterogeneous clinical presentations. Notably, early-onset diabetes has been associated with more aggressive disease progression and complications [45], underscoring the clinical importance of refining this cluster beyond age alone. These findings highlight that severe forms have strong pathophysiological anchors, whereas mild forms are more diffuse and may represent transitional or overlapping states influenced by environmental, lifestyle, and demographic factors.

The successful replication of severe T2D subtypes underscores the translational potential of clustering for patient stratification at diagnosis. Subtype assignment can guide pharmacotherapy: SIDD patients benefit from early insulin or insulin secretagogues; SIRD patients from GLP-1 receptor agonists or SGLT2 inhibitors; MOD patients from weight-centric therapies, including incretin-based treatments or bariatric surgery; MARD patients from simple regimens such as metformin with low hypoglycaemia risk; and MEOD patients from intensive early lifestyle or incretin-based interventions [46,47,48]. Subtyping can also inform complication surveillance, as SIDD carries heightened microvascular risk while SIRD is linked to nephropathy and cardiovascular complications [2,44,49]. Clinically, patients within overlap zones may face uncertainty in therapeutic allocation, reinforcing the need to integrate molecular, genetic, or longitudinal biomarkers to sharpen cluster boundaries [8,18].

Our study has several strengths. Unlike prior clustering work that validated subtypes within the same cohort [2] or derived mechanistic axes from genetic data [19], we directly tested the portability of clinical subtypes across independent cohorts. By applying supervised ML models to predict cluster membership in a mixed-ethnicity, newly diagnosed cohort, we assessed the real-world feasibility of subtype classification at diagnosis. Furthermore, reproducibility was rigorously quantified using ARI and FMI, providing a rigorous measure of similarity across clustering approaches. Using only five key variables (FBG, FSI, HbA1c, BMI, age at diagnosis) allowed clustering to focus on etiological influences, and despite differences in ethnicity, disease duration, complications, and medication use between cohorts, the same five T2D subtypes emerged, with variation only in individual cluster membership. Although sex-specific differences in insulin sensitivity and fat distribution are well documented [50,51] sex had no effect in the training dataset [17], though other untested confounders remain a study limitation. Our training dataset was smaller and restricted to a homogeneous Emirati population compared to the larger, multi-ethnic prediction dataset which may lead to underfitting in ML models. Reliance on cross-sectional measures such as FSI and FBG may not capture temporal dynamics in insulin resistance and β-cell function, although exploratory analyses suggest that snapshot HOMA values correlate with longitudinal averages in steady states.

The instability of mild clusters suggests that clinical variables alone may be insufficient for robust patient stratification. Multi-omic integration—incorporating genetics, proteomics, and metabolomics—has shown promise in refining T2D subtypes [8,18,23]. Polygenic partitioning, for example, improves the mechanistic resolution of T2D heterogeneity [19]. Environmental and lifestyle factors, including diet, adiposity, and physical activity, should also be integrated to account for non-genetic influences on phenotype [8,18,52,53]. Longitudinal designs will be essential to distinguish transitional from stable phenotypes [54] and to evaluate whether cluster assignment predicts treatment response and complication trajectories.

## 5. Conclusions

In summary, our study confirms that severe T2D subtypes (SIRD and SIDD) are reproducible across cohorts and clustering methods, whereas mild forms (MARD, MOD, and MEOD) exhibit weaker stability due to phenotypic overlap. This underscores both the promise and the limitations of clustering based on clinical variables alone. Improving reproducibility—particularly for mild clusters—will require integration of genetic, molecular, and longitudinal data. Ultimately, refining subtype classification has the potential to advance precision endocrinology by guiding therapeutic choices, monitoring complications, and tailoring lifestyle interventions.

## Figures and Tables

**Table 1:** The pathophysiological characteristics of the training and prediction datasets.

Variables	Training Dataset (*N* = 348)	Prediction Dataset (*N* = 586)	*p*-Value
Age at diagnosis (years)	41.92 (10.65)	46.32 (9.27)	**<0.001**
Body mass index (kg/m^2^)	31.27 (5.7)	31.54 (6.04)	0.247
Fasting blood glucose (mg/dL)	146.86 (52.47)	133 (44.16)	**<0.001**
HbA1c (%)	7.63 (1.72)	7 (1.33)	**<0.001**
Fasting serum insulin (µIU/nmol)	14.04 (10.67)	18.04 (10.74)	**<0.001**
Duration of type 2 diabetes (years)	14.42 (8.14)	3.67 (2.9)	**<0.001**

Data is shown as Mean (±standard deviation). *p*-value was determined using Mann–Whitney *U* test. *p* < 0.05-significant (bold).

**Table 2:** Crosstabulation between T2D subtypes generated by direct clustering versus cluster-based classification using ML predictive models in prediction dataset (*N* = 586) for three scenarios (1, 2 and 4) of exploratory variables.

		Supervised Cluster-Based Classification Using ML Predictive Models					
		SIRD	SIDD	MARD	MOD	MEOD	Total
**Direct unsupervised clustering**	*Scenario 1*: FSI, FBG, and BMI						
	SIRD	46	2	28	5	0	81
	SIDD	0	17	0	1	0	18
	MARD	0	15	8	0	38	61
	MOD	0	1	36	96	6	139
	MEOD	0	0	78	0	209	287
	Total	46	35	150	102	253	586
	*Scenario 2*: FSI, FBG, and BMI and age at diagnosis						
	SIRD	56	0	0	2	0	58
	SIDD	3	28	1	1	0	33
	MARD	1	3	185	0	21	210
	MOD	20	0	66	39	0	125
	MEOD	5	1	17	43	94	160
	Total	85	32	269	85	115	586
	*Scenario 4*: FSI, FBG, and BMI, HbA1c and age at diagnosis						
	SIRD	62	0	2	0	0	64
	SIDD	4	38	1	1	1	45
	MARD	4	1	104	30	94	233
	MOD	7	1	4	79	0	91
	MEOD	0	2	90	9	52	153
	Total	77	42	201	119	147	586

FSI: fasting serum insulin; FBG: fasting blood glucose; BMI: body mass index; SIRD: severe insulin-resistant diabetes; SIDD: severe insulin-deficient diabetes; MARD: mild age-related diabetes; MOD: mild obesity-related diabetes; MEOD: mild early-onset diabetes. The *scenarios* are highlighted in Grey.

**Table 3:** Crosstabulation between T2D subtypes generated by direct clustering versus cluster-based classification using ML predictive models in prediction dataset (*N* = 586) for *Scenario 3* (FSI, FBG, and BMI and HbA1c).

	Supervised Cluster-Based Classification Using ML Predictive Models				
**Direct unsupervised clustering**		**SIRD**	**SIDD**	**Mixed**	**Total**
	SIDD	6	51	1	58
	SIRD	136	1	81	218
	Mixed	0	6	304	310
	Total	142	58	386	586

SIRD: severe insulin-resistant diabetes; SIDD: severe insulin-deficient diabetes; MARD: mild age-related diabetes; MOD: mild obesity-related diabetes; MEOD: mild early-onset diabetes.

## Data Availability

The raw data supporting the conclusions of this article will be made available by the authors on request.

## Supplementary Materials

The following supporting information can be downloaded at https://www.mdpi.com/article/10.3390/jcm14186548/s1, Method S1: Statistical basis for exclusion of HOMA indices as explanatory variables; Method S2: Calculation of the Adjusted Rand Index (ARI); Method S3: Calculation of Confidence intervals; Table S1: The Adjusted Rand Index (ARI) between T2D clusters generated by direct clustering versus cluster-based classification using machine learning predictive models in prediction dataset, employing various combinations of exploratory variables; Table S2: The Fowlkes-Mallows Index (FMI) between T2D clusters generated by direct clustering versus cluster-based classification using machine learning predictive models in prediction dataset, employing combinations of exploratory variables; Table S3: Comparison of characteristics between direct and predicted clusters using five exploratory variables.
