# Supplementary material for: Appraisal of Clinical Explanatory Variables in Subtyping of Type 2 Diabetes Using Machine Learning Models
Source: J Clin Med. 2025 Sep 17;14(18):6548. doi: 10.3390/jcm14186548 (PMC12470861; doi:10.3390/jcm14186548)
Supplement: Supplementary file 1 [file jcm-14-06548-s001.zip › jcm-3832851-supplementary.pdf]

Supplementary Material

Supplementary Method S1: Statistical basis for exclusion of HOMA indices as explanatory variables

1. **Variance Inflation Factor (VIF) Analysis**

VIF measures how much the variance of a regression coefficient is inflated due to collinearity with other predictors. VIF was calculated with the formula:

$$\mathrm{VIF} = \frac{1}{1 - \mathrm{R}_\mathrm{i}^2}$$

Where R_i^2 , is a coefficient of determination from the regression.
Calculations:

a) Regression coefficient:

Model Summary				
Model	R	R Square	Adjusted R Square	Std. Error of the Estimate
1	.964[a]	.929	.929	1.27394
a. Predictors: (Constant), Fasting Blood Glucose, Fasting Serum Insulin				

b) Variance:

		Fasting Serum Insulin	Fasting Blood Glucose	HOMA-IR
N	Valid	348	348	348
	Missing	0	0	0
Variance		113.929	2753.282	22.861

c) VIF:

$$\mathrm{VIF} = \frac{1}{1 - 0.929} = 14.1$$

A VIF > 5 is considered problematic and indicates multicollinearity.

2. **Correlation Matrix Evidence**

	Fasting serum insulin	Fasting blood glucose	HOMA-IR
Fasting serum insulin	1	.117*	.889**
Fasting blood glucose		1	.473**
HOMA-IR			1

*Correlation is significant at the 0.05 level (2-tailed).
**Correlation is significant at the 0.01 level (2-tailed).
Correlations > 0.85 indicate redundancy.

Supplementary Method S2: Calculation of the Adjusted Rand Index (ARI)

ARI, a metric used to measure the similarity between two data clustering's was calculated as:

$$ARI = \frac{RI - E}{Max\ RI - E}$$

Where RI is Rand index value, E is the expected value of the Rand index for random clusters, and Max RI is the maximum achievable value of the Rand index.

Step 1: The Rand Index (*RI*) is calculated as:

$$RI = \frac{a + d}{a + b + c + d}$$

Where,

a: The number of pairs of points that are in the same cluster in both cluster sets.

b: The number of pairs of points that are in the same cluster in Set 1 but in different clusters in Set 2.

c: The number of pairs of points that are in different clusters in Set 1 but in the same cluster in Set 2.

d: The number of pairs of points that are in different clusters in both sets.

Step 2: Expected RI (E) is calculated as:

$$E = \frac{\binom{n_1}{2} + \binom{n_2}{2} + \cdots \binom{n_k}{2}}{\binom{N}{2}}$$

Where $n_1, n_2, \ldots, n_k$ are sizes of the clusters in set 1 and set 2; and N is the total number of the data points.

Step 3: Max RI is calculated as:

$$Max\ RI = \frac{\binom{N}{2}}{\binom{N}{2}}$$

Supplementary Method S3: Calculation of Confidence intervals

The 95% CI for both ARI and FMI were calculated as

Step 1: Fisher z-transform

$$z = \frac{1}{2}\ln\left(\frac{(1+r)}{(1-r)}\right)$$

Where r is ARI or FMI values

Step 2: Standard error in z-space

$$SE_z = \frac{1}{\sqrt{n-3}}$$

Where n is the sample size

Step 3: 95%CI in z-space

$$z_{95CI} = z \pm 1.96SE_z$$

Supplementary results

Supplementary Table S1: The Adjusted Rand Index (ARI) between T2D clusters generated by direct clustering versus cluster-based classification using machine learning predictive models in prediction dataset, employing various combinations of exploratory variables.

	**Adjusted Rand Index [95%CI]**			
	Scenario 1 (FSI, FBG, BMI)	***Scenario 2*** (FSI, FBG, BMI and Age at diagnosis)	***Scenario 3*** (FSI, FBG, BMI, and HbA1c)	***Scenario 4*** (FSI, FBG, BMI, HbA1c and Age at diagnosis)
**SIRD**	0.91 [0.9 -0.92]	0.92 [0.91-0.94]	0.81 [0.78-0.84]	0.96 [0.93-0.95]
**SIDD**	0.95 [0.94 – 0.96]	0.98 [0.97-0.98]	0.96 [0.95-0.97]	0.97 [0.96-0.97]
**MARD**	0.63 [0.58 – 0.67]	0.62 [0.56-0.66]	NA	0.42 [0.36-0.49]
**MOD**	0.86 [0.84 – 0.88]	0.71 [0.67-0.75]	NA	0.80 [0.77-0.83]
**MEOD**	-0.28 [-0.36 (-0.21)]	0.79 [0.75-0.82]	NA	0.54 [0.48-0.60]
**Mixed**	NA	NA	0.51 [0.45-0.57]	NA

CI: Confidence Interval; FSI: Fasting Serum Insulin; FBG: Fasting Blood Glucose; BMI: Body Mass Index; SIRD: Severe Insulin Resistant Diabetes; SIDD: Severe Insulin Deficient Diabetes; MARD: Mild Age-related Diabetes; MOD: Mild Obesity-related Diabetes; MEOD: Mild Early Onset Diabetes; NA: Not applicable.

Supplementary Table S2: The Fowlkes-Mallows Index (FMI) between T2D clusters generated by direct clustering versus cluster-based classification using machine learning predictive models in prediction dataset, employing combinations of exploratory variables.

	**Fowlkes-Mallows Index [95% CI]**			
	Scenario 1 (FSI, FBG, BMI)	***Scenario 2*** (FSI, FBG, BMI and Age at diagnosis)	***Scenario 3*** (FSI, FBG, BMI, and HbA1c)	***Scenario 4*** (FSI, FBG, BMI, HbA1c and Age at diagnosis)
**SIRD**	0.75 [0.71-0.79]	0.80 [0.77-0.82]	0.77 [0.74-0.80]	0.88 [0.86-0.90]
**SIDD**	0.68 [0.63-0.72]	0.85 [0.82-0.87]	0.88 [0.86-0.90]	0.87 [0.85-0.89]
**MARD**	0.08 [0.03-0.16]	0.78 [0.74-0.81]	NA	0.48 [0.42-0.54]
**MOD**	0.81 [0.78-0.83]	0.38 [0.31-0.45]	NA	0.76 [0.72-0.79]
**MEOD**	0.78 [0.74-0.81]	0.69 [0.64-0.73]	NA	0.35 [0.27-0.42]
**Mixed**	NA	NA	0.88 [0.86-0.90]	NA

CI: Confidence Interval; FSI: Fasting Serum Insulin; FBG: Fasting Blood Glucose; BMI: Body Mass Index; SIRD: Severe Insulin Resistant Diabetes; SIDD: Severe Insulin Deficient Diabetes; MARD: Mild Age-related Diabetes; MOD: Mild Obesity-related Diabetes; MEOD: Mild Early Onset Diabetes; NA: Not applicable.

Supplementary Table S3: Comparison of characteristics between direct and predicted clusters using five exploratory variables.

**Clusters**		**Unsupervised direct clustering**	**Supervised cluster-based prediction**	**P-value**
**SIRD**	Number of patients (*n*)	64	77	
	Age (years)	47.89 (8.95)	47.45 (9.69)	0.7796
	Age of diagnosis (years)	45.02 (8.16)	44.14 (8.96)	0.5429
	Duration of T2D (years)	2.88 (2.47)	3.31 (2.91)	0.3469
	BMI (kg/m^2)	34.66 (5.35)	34.64 (5.3)	0.9822
	FBG (mg/dL)	132.11 (31.24)	140.26 (46.36)	0.2277
	FSI (µIU/nmol)	40.88 (9.1)	39.6 (8.86)	0.3965
	HbA1c (%)	6.96 (1.1)	7.14 (1.28)	0.3729
	HOMA-IR	13.47 (4.76)	13.77 (5.41)	0.7545
	HOMA-B (%)	255.58 (137.79)	244.78 (147.56)	0.4930
**SIDD**	Number of patients (*n*)	45	42	
	Age (years)	48.96 (8.25)	47.9 (9.27)	0.5741
	Age of diagnosis (years)	44.38 (8.1)	44 (9.23)	0.8385
	Duration of T2D (years)	4.58 (3.47)	3.9 (2.95)	0.3292
	BMI (kg/m^2)	30.84 (5.06)	30.13 (5.36)	0.5268
	FBG (mg/dL)	241 (58.78)	235.05 (61.94)	0.6469
	FSI (µIU/nmol)	16.03 (9.25)	13.77 (6.41)	0.1919
	HbA1c (%)	10.13 (1.49)	10.1 (1.6)	0.9281
	HOMA-IR	9.51 (6.38)	7.82 (3.82)	0.1374
	HOMA-B (%)	36.25 (22.65)	35.04 (24.7)	0.8122
**MARD**	Number of patients (*n*)	233	201	
	Age (years)	57.7 (4.74)	49.91 (9.38)	**<0.001**
	Age of diagnosis (years)	54.24 (4.77)	46.21 (9.35)	**<0.001**
	Duration of T2D (years)	3.46 (2.69)	3.7 (2.79)	0.3628
	BMI (kg/m^2)	29.39 (4.26)	29.59 (3.02)	0.5699
	FBG (mg/dL)	122.13 (24.95)	117.5 (20.68)	**0.0377**
	FSI (µIU/nmol)	6.67 (0.77)	6.59 (0.73)	0.2696
	HbA1c (%)	14.15 (6.36)	18 (4.5)	**<0.001**
	HOMA-IR	4.23 (1.98)	5.28 (1.74)	**<0.001**
	HOMA-B (%)	106.22 (82.97)	136.58 (69.47)	**0.001**
**MOD**	Number of patients (*n*)	91	119	
	Age (years)	44.43 (9)	48.89 (9.84)	**0.0005**
	Age of diagnosis (years)	41.02 (8.09)	45.38 (9.22)	**0.0004**
	Duration of T2D (years)	3.41 (3.04)	3.51 (3.01)	0.8125
	BMI (kg/m^2)	40.42 (4.67)	39.39 (4.54)	0.1091
	FBG (mg/dL)	131.12 (34.45)	127.16 (30.38)	0.3782
	FSI (µIU/nmol)	6.92 (1.06)	6.88 (1.04)	0.78.44
	HbA1c (%)	20.59 (6.02)	17.65 (6.13)	**0.0006**
	HOMA-IR	6.78 (3.08)	5.63 (2.73)	**0.0047**
	HOMA-B (%)	134.07 (67.8)	118.79 (63.41)	**0.0227**
**MEOD**	Number of patients (*n*)	153	147	
	Age (years)	42.76 (6.7)	52.95 (8.52)	**<0.001**

	Age of diagnosis (years)	38.54 (5.88)	49.05 (8.82)	**0.0259**
	Duration of T2D (years)	4.22 (2.97)	3.89 (2.93)	**0.3336**
	BMI (kg/m^2)	28.44 (3.49)	26.63 (2.95)	**<0.0001**
	FBG (mg/dL)	119.22 (24.88)	127.16 (30.38)	**0.0137**
	FSI (µIU/nmol)	6.66 (0.94)	6.72 (0.89)	0.5710
	HbA1c (%)	13.49 (5.75)	8.33 (2.56)	**<0.001**
	HOMA-IR	4.01 (1.92)	2.63 (1.05)	**<0.001**
	HOMA-B (%)	101.05 (59.56)	57.77 (30.05)	**<0.001**

T2D: Type 2 diabetes; BMI: body mass index; FBG: fasting blood glucose; FSI: fasting serum insulin; HbA1c: glycated haemoglobin; HOMA-IR: homeostatic model assessment of insulin resistance; HOMA-B: homeostatic model assessment of β-cell dysfunction; SIRD: severe insulin resistant diabetes; SIDD: severe insulin deficient diabetes; MARD: mild age-related diabetes; MOD: mild obesity-related diabetes; MEOD: mild early onset diabetes.

Data is shown as Mean (± standard deviation).

P-value was determined using Mann-Whitney *U* test. P<0.05-significant (bold).
